# Leptin receptor gene deficiency minimally affects osseointegration in rats

**DOI:** 10.1038/s41598-023-42379-5

**Published:** 2023-09-20

**Authors:** Martina Jolic, Krisztina Ruscsák, Lena Emanuelsson, Birgitta Norlindh, Peter Thomsen, Furqan A. Shah, Anders Palmquist

**Affiliations:** Department of Biomaterials, Institute of Clinical Sciences, Sahlgrenska Academy, University of Gothenburg, Göteborg, Sweden

**Keywords:** Bone, Implants, Bone quality and biomechanics, Type 2 diabetes, Obesity

## Abstract

Metabolic syndrome represents a cluster of conditions such as obesity, hyperglycaemia, dyslipidaemia, and hypertension that can lead to type 2 diabetes mellitus and/or cardiovascular disease. Here, we investigated the influence of obesity and hyperglycaemia on osseointegration using a novel, leptin receptor-deficient animal model, the Lund MetS rat. Machined titanium implants were installed in the tibias of animals with normal leptin receptor (LepR^+/+^) and those harbouring congenic leptin receptor deficiency (LepR^−/−^) and were left to heal for 28 days. Extensive evaluation of osseointegration was performed using removal torque measurements, X-ray micro-computed tomography, quantitative backscattered electron imaging, Raman spectroscopy, gene expression analysis, qualitative histology, and histomorphometry. Here, we found comparable osseointegration potential at 28 days following implant placement in LepR^−/−^ and LepR^+/+^ rats. However, the low bone volume within the implant threads, higher bone-to-implant contact, and comparable biomechanical stability of the implants point towards changed bone formation and/or remodelling in LepR^−/−^ rats. These findings are corroborated by differences in the carbonate-to-phosphate ratio of native bone measured using Raman spectroscopy. Observations of hypermineralised cartilage islands and increased mineralisation heterogeneity in native bone confirm the delayed skeletal development of LepR^−/−^ rats. Gene expression analyses reveal comparable patterns between LepR^−/−^ and LepR^+/+^ animals, suggesting that peri-implant bone has reached equilibrium in healing and/or remodelling between the animal groups.

## Introduction

Metal implants are an important treatment component in the dental, maxillofacial, and orthopaedic fields. Their effectiveness stems from osseointegration—direct contact between the living bone and the metal implant without intervening soft tissue^1^. However, while rare, failures of osseointegration can lead to significant negative consequences on the individual’s health and socioeconomic status. Obesity affects 13% of adults^2^, and has gathered increasing attention with accumulating evidence linking obesity to compromised bone quality, coupled with osteoporosis and increased risk of bone fractures in the elderly demographic^3^. Additionally, 10% of adults are living with diabetes, with half of those cases going undiagnosed, and another 10% exhibiting impaired glucose tolerance^4^. These metabolic disorders can further complicate bone health and impede healing processes^5^. Considering these factors together with the ageing population^6^, it is likely that the prevalence of individuals with underlying adverse medical conditions relying on implants to maintain a normal quality of life will increase.

Implant anchorage and the overall longevity of metal implants are highly dependent on bone mechanochemical properties^7^. The hierarchical structure of bone consists of organic (i.e., proteins, mainly collagen type I), inorganic (i.e., carbonated apatite), and cellular constituents^8^. As such, overall bone quality can be affected by disorders affecting bone mineralisation, collagen, and/or bone healing, remodelling, and turnover^9^. Bone homeostasis is maintained by the highly coordinated actions of bone cells (i.e., osteoblasts, osteoclasts, and osteocytes) through various signalling pathways^10^. An increased risk of vertebral fractures has been linked to higher blood concentrations of sclerostin^11^, an important regulator of the canonical Wnt/β-catenin signalling pathway, which has been shown to inhibit the expression of bone turnover markers in type 2 diabetes mellitus patients^12,13^. Sclerostin can also indirectly influence bone resorption by promoting osteoclastic activity through control of RANKL/OPG (receptor activator of nuclear factor-kappa B ligand/osteoprotegerin) levels^14^. In proinflammatory and inflammatory conditions, osteoclastic differentiation can also be initiated by TNFa (tumour necrosis factor alpha) acting together with IL-1 (interleukin-1)^15^. One such condition is obesity, where elevated levels of TNFa in adipose tissue point to an underlying chronic inflammation^16^. Although obese individuals usually present with high bone mineral density (BMD), conventionally perceived as a sign of good bone strength and low risk of fractures^17^, studies in various animal models have demonstrated the detrimental effects of obesity on bone quality^18^, regeneration^19^, and osseointegration^20,21^. Hyperglycaemia, another potential contraindication for successful implant treatment, has been shown to reduce angiogenesis and compromise healing in critical size calvarial defects^22^. Even though hyperglycaemia has not been irrefutably linked to poor dental implant survival, marginal bone loss is almost consistently reported^23^ in clinical studies. The level of osseointegration in ex vivo samples is typically determined by measuring the extent of the bone-implant contact (BIC) in histological preparations^24^, with reduced BIC often reported in animal models of diabetes^25–28^.

Metabolic syndrome is a complex condition exhibiting features such as hyperglycaemia, dyslipidaemia, central obesity, and hypertension that can lead to type 2 diabetes mellitus and/or cardiovascular disease^29^. In this work, we used the Lund MetS rat, which mimics metabolic syndrome in humans by developing severe obesity, hyperglycaemia, and high blood pressure in addition to elevated cholesterol and triglyceride levels due to a leptin receptor mutation^30^. Leptin, a cytokine-like hormone secreted by adipocytes, was first described as a product of the obese gene that regulates body weight and energy expenditure^31^. Homozygous mutations of the leptin receptor have been shown to lead to severe early-onset obesity^32,33^, and disorders involving both leptin and leptin receptor have been implicated in immunity, haematopoiesis, angiogenesis, reproduction, and bone production^34^.

Using a novel animal model harbouring leptin receptor deficiency (LepR^−/−^)^35^, closely mimicking the human metabolic syndrome milieu, together with severe developmental impairment^36^, we investigated the influence of hyperglycaemia and obesity on osseointegration. The animal model and extent of osseointegration were evaluated using qualitative histology and histomorphometry, removal torque (RTQ) measurements assessing the biomechanical stability of the implants, X-ray micro-computed tomography (micro-CT), quantitative backscattered electron imaging (qBEI), Raman spectroscopy, and gene expression analysis.

## Results

### Animal model evaluation

**Figure 1:**
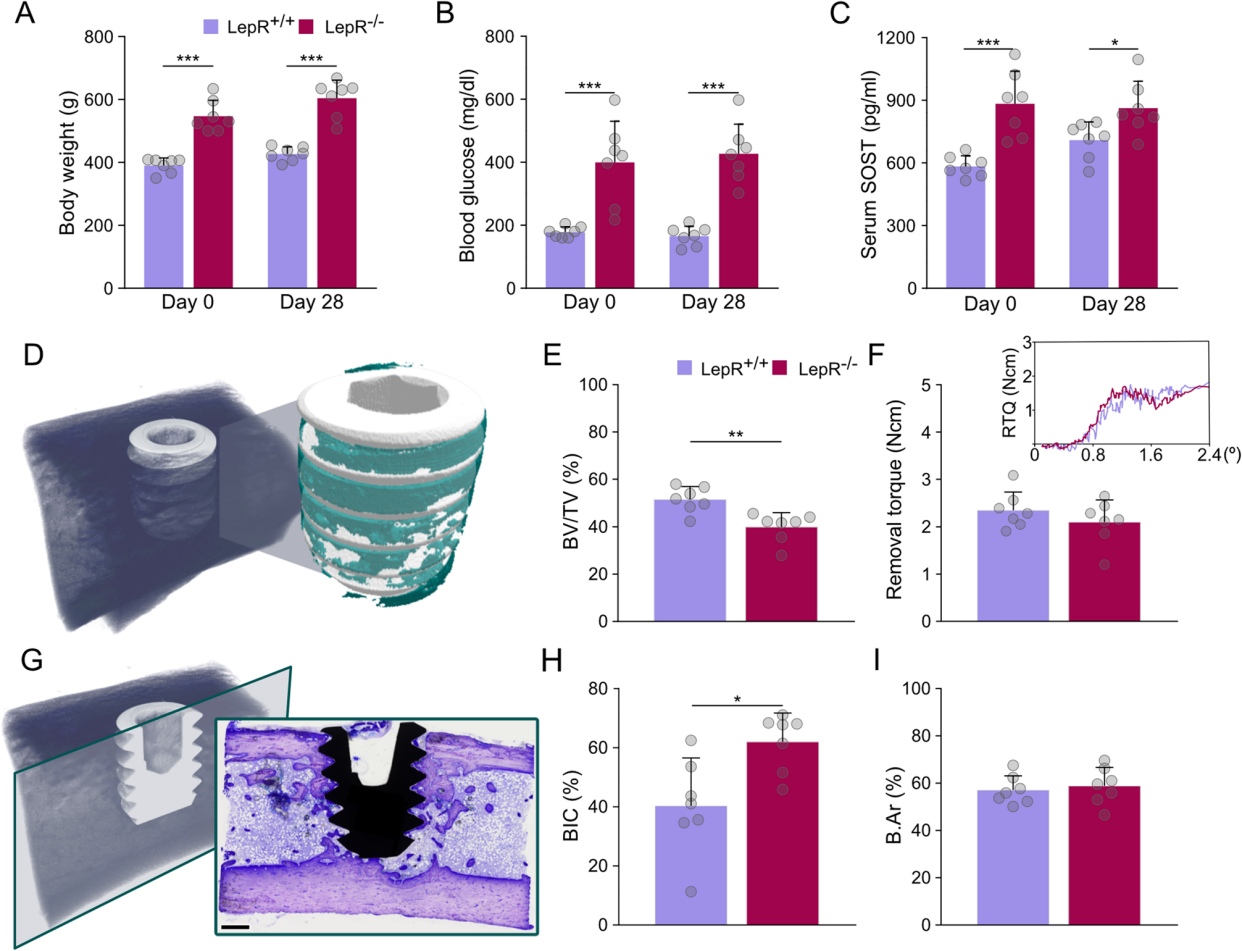
Animal model characterisation and assessment of osseointegration. (**A**) Body weight of LepR^+/+^ and LepR^−/−^ animals on Day 0 and Day 28. (**B**) Blood glucose levels of LepR^+/+^ and LepR^−/−^ animals on Day 0 and Day 28. (**C**) Serum sclerostin concentrations on Day 0 and Day 28. (**D**) Region of interest selected for measurements of bone volume using micro-CT. (**E**) Bone volume measured in the total volume of interest (BV/TV) using micro-CT. (**F**) Biomechanical stability of the implants measured by removal torque (RTQ). Inset: average load deformation curves (applied force vs. angular deformation). (**G**) Example of the histological section made in the sagittal plane of the bone. Scale bar = 500 µm. (**H**) Bone-implant contact (BIC). (**I**) Bone area (B.Ar) present within the implant threads. *p < 0.05, **p < 0.01, ***p < 0.001*.*

**Figure 2:**
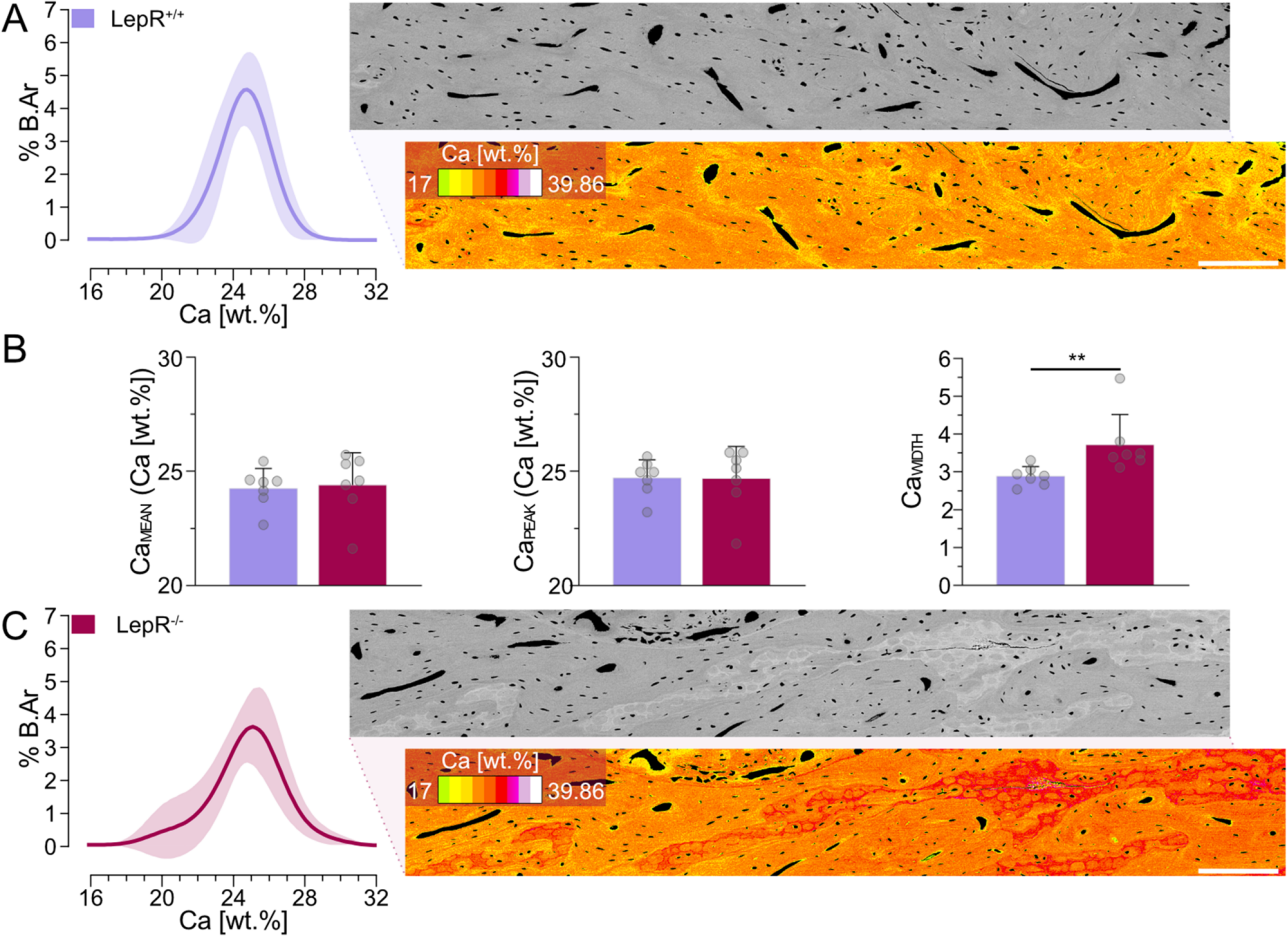
Bone mineral density distribution (BMDD). (**A**) Left: BMDD curve generated from the histogram data of native bone in LepR^+/+^ animals (*n* = 7). Right: representative qBEI image (with and without 16-level lookup table). Scale bar = 200 µm. (**B**) BMDD parameters. From left to right: Ca_MEAN_, Ca_PEAK_, and Ca_WIDTH_. (**C**) Left: BMDD curve generated from the histogram data of native bone in LepR^−/−^ animals (*n* = 7). Right: representative qBEI image (with and without 16-level lookup table). Scale bar = 200 µm. **p < 0.01.

Body weight and blood glucose levels were monitored in all animals during the 28-day healing period. LepR^−/−^ rats were significantly heavier, weighing 547.1 ± 50.2 g on Day 0 (p < 0.001) and 604.1 ± 57.8 g on Day 28 (p < 0.001), while LepR^+/+^ rats weighed 390.6 ± 32.6 g on Day 0 and 427.0 ± 22.5 g on Day 28 (Fig. 1A). Higher blood glucose levels were measured in LepR^−/−^ animals. On Day 0, blood glucose levels in the LepR^−/−^ rats were 399.6 ± 130.5 mg/dl (p < 0.001) and 177.9 ± 17.3 mg/dl in LepR^+/+^ rats. On Day 28, in LepR^−/−^ animals, the blood glucose concentration was 426.9 ± 94.3 mg/dl (p < 0.001), and in LepR^+/+^ animals, it was 165.9 ± 31.1 mg/dl (Fig. 1B). The concentration of sclerostin was measured using enzyme-linked immunosorbent assay (ELISA) in the serum of animals, where a ~51% higher concentration was detected in LepR^−/−^ rats on Day 0 (p < 0.001), while only a ~22% higher concentration was measured on Day 28 (p < 0.05) (Fig. 1C). Bone mineral density distribution (BMDD) was evaluated in the native bone of LepR^+/+^ and LepR^−/−^ rats using three parameters (Ca_MEAN_, Ca_PEAK_, and Ca_WIDTH_) as described by Roschger et al.^37^ (Fig. 2A–C). No differences in Ca_MEAN_ and Ca_PEAK_ were observed between the LepR^+/+^ and LepR^−/−^ rats. More heterogeneous BMDD was detected in the LepR^−/−^ animals, where the Ca_WIDTH_ was 3.5 ± 0.6 (p < 0.01), while increased homogeneity in LepR^+/+^ rats resulted in a Ca_WIDTH_ of 2.7 ± 0.24.

### Assessment of osseointegration

The biomechanical stability of implants was assessed using continuous rotational force applied to the exposed implant head at the experimental endpoint^38^. The RTQ values were 2.1 ± 0.5 Ncm in LepR^−/−^ animals and 2.4 ± 0.4 Ncm in LepR^+/+^ animals (Fig. 1F). Bone volume measured within the region of interest (ROI) was 23% lower in LepR^−/−^ animals (p < 0.01) (BV/TV, i.e., bone volume/total volume) (Fig. 1D, E), even though the BIC of 62.06 ± 9.68% was significantly higher in LepR^−/−^ rats (p < 0.05) when compared to the BIC of 40.34 ± 16.21% measured in LepR^+/+^ rats. Bone area (B.Ar) within the implant threads revealed no significant difference between the LepR^+/+^ and LepR^−/−^ animals (Fig. 1G–I).

### Raman spectroscopy

**Figure 3:**
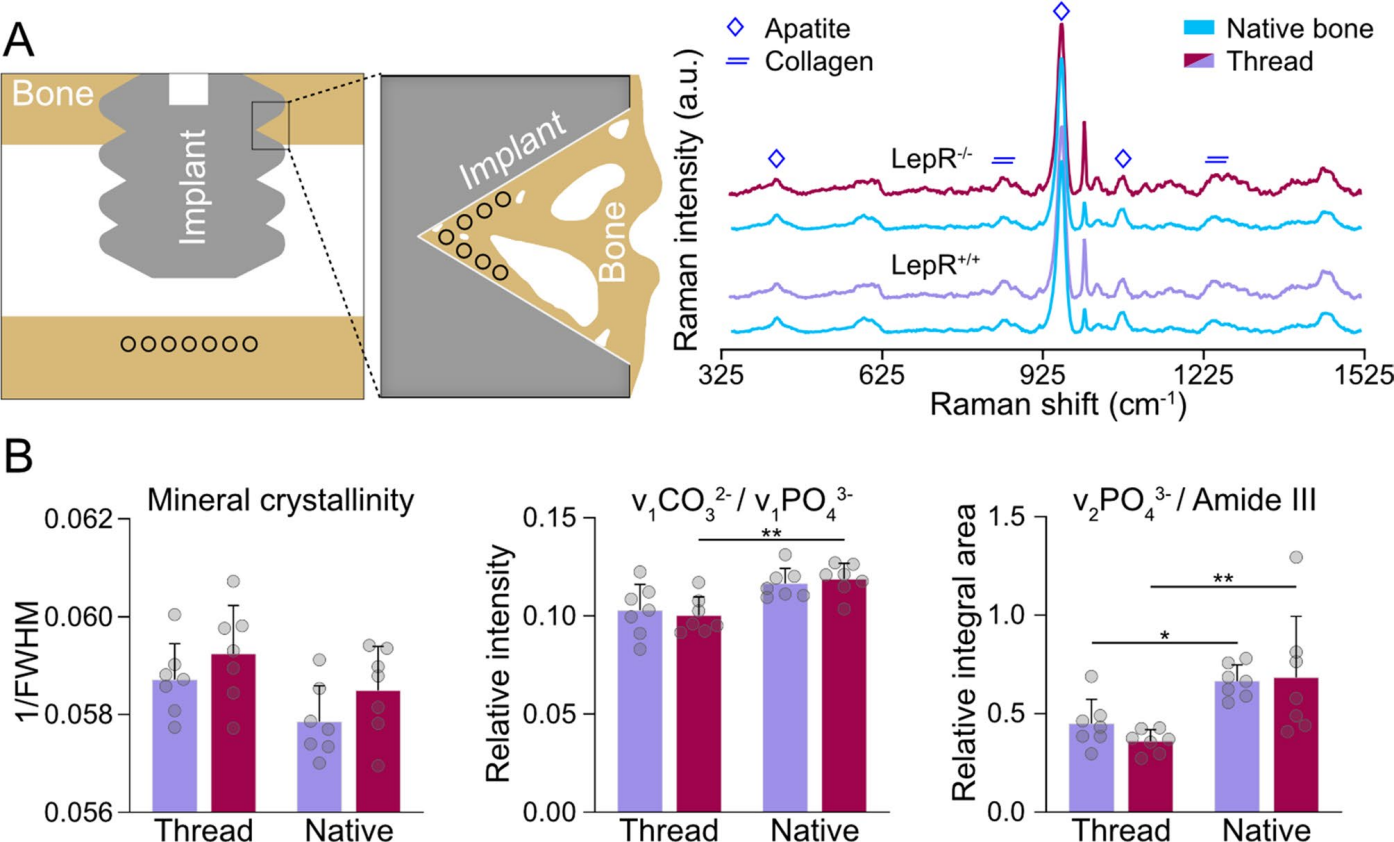
Chemical composition of bone. (**A**) Left: schematic representation of the Raman measurement points (black circles) of bone within the implant threads and native bone. Right: Raman spectra of bone within the implant threads and in the native bone of LepR^−/−^ (*n* = 7) and LepR^+/+^ (*n* = 7) animals showing the characteristic peaks for apatite (ν_2_PO_4_^3−^ at ~420 to 470, ν_1_PO_4_^3−^ at ~959, and ν_1_CO_3_^2−^ ~ 1070 cm^−1^) and collagen (proline at ~855, hydroxyproline at ~876, and amide III at ~1240 to 1270 cm^−1^). (**B**) From left to right: mineral crystallinity, carbonate-to-phosphate ratios, and mineral-to-matrix ratios of bone within the implant threads and native bone. *p < 0.05 and **p < 0.01*.*

The Raman spectra of bone within the implant threads and in the native bone of LepR^+/+^ and LepR^−/−^ animals displayed typical spectral features associated with the organic and inorganic phases of bone (Fig. 3A). Mineral crystallinity, taken as the inverse full-width at half-maximum (1/FWHM) of the ν_1_PO_4_^3−^ peak (~959 cm^−1^), was found to be similar in both LepR^+/+^ and LepR^−/−^ rats. In the native bone, the carbonate-to-phosphate ratio, calculated as the intensity ratio of the ν_1_CO_3_^2−^ and ν_1_PO_4_^3−^ peaks (~1070 cm^−1^ and ~959 cm^−1^, respectively), was consistently higher, with a significant difference observed only in LepR^−/−^ animals (p < 0.001). The mineral-to-matrix ratio, calculated as the ratio of the integral areas of ν_2_PO_4_^3−^ and amide III bands (~420 to 470 cm^−1^ and ~1240 to 1270 cm^−1^, respectively), indicated an almost 90% higher mineralisation in the native bone of the LepR^−/−^ animals (p < 0.01) and an approximately 48% higher mineralisation in the native bone of LepR^+/+^ animals (p < 0.05) compared to the bone within the implant threads (Fig. 3B).

### Gene expression analysis

**Figure 4:**
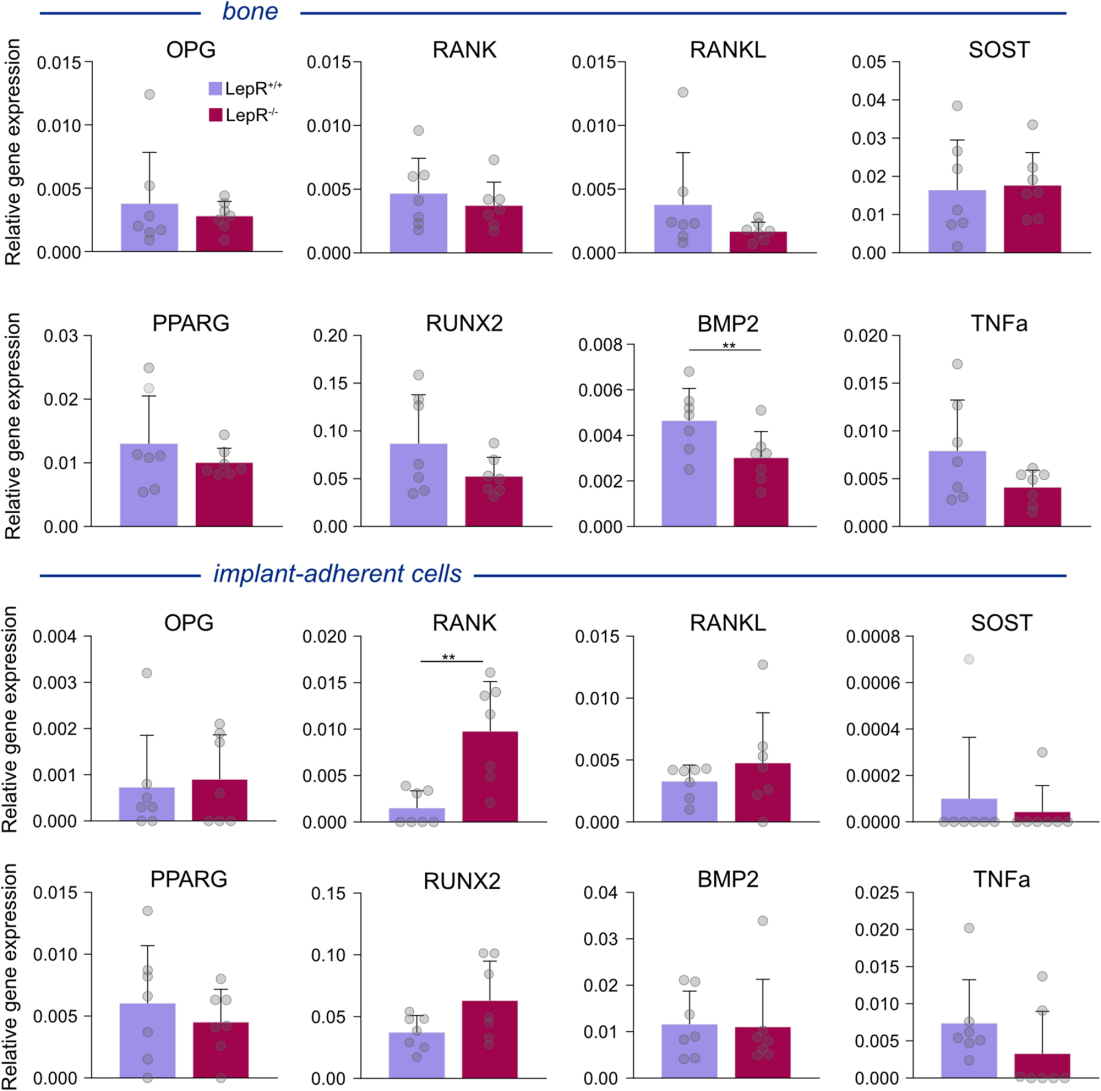
Gene expression analysis of bone and implant-adherent cells. Relative expression of genes coding for sclerostin (SOST), receptor activator of nuclear factor-kappa B (RANK), receptor activator of nuclear factor-kappa B ligand (RANKL), osteoprotegerin (OPG), peroxisome proliferator-activated receptor gamma (PPARG), Runt-related transcription factor 2 (RUNX2), bone morphogenetic protein 2 (BMP2), and tumour necrosis factor alpha (TNFa) was analysed for both LepR^+/+^ (*n* = 7) and LepR^−/−^ animals (*n* = 7). *p < 0.05, **p < 0.01.

Relative gene expression was evaluated in peri-implant bone and implant-adherent cells using droplet digital PCR (ddPCR) technology (Fig. 4). Gene expression patterns were generally comparable between the LepR^+/+^ and LepR^−/−^ animals. In peri-implant bone, the relative expression of bone morphogenetic protein 2 (BMP2) was significantly higher in LepR^+/+^ rats (p < 0.05). In samples of implant-adherent cells, the relative gene expression of receptor activator of nuclear factor-kappa B (RANK) was significantly higher in LepR^−/−^ animals (p < 0.01).

### Histological evaluation

**Figure 5:**
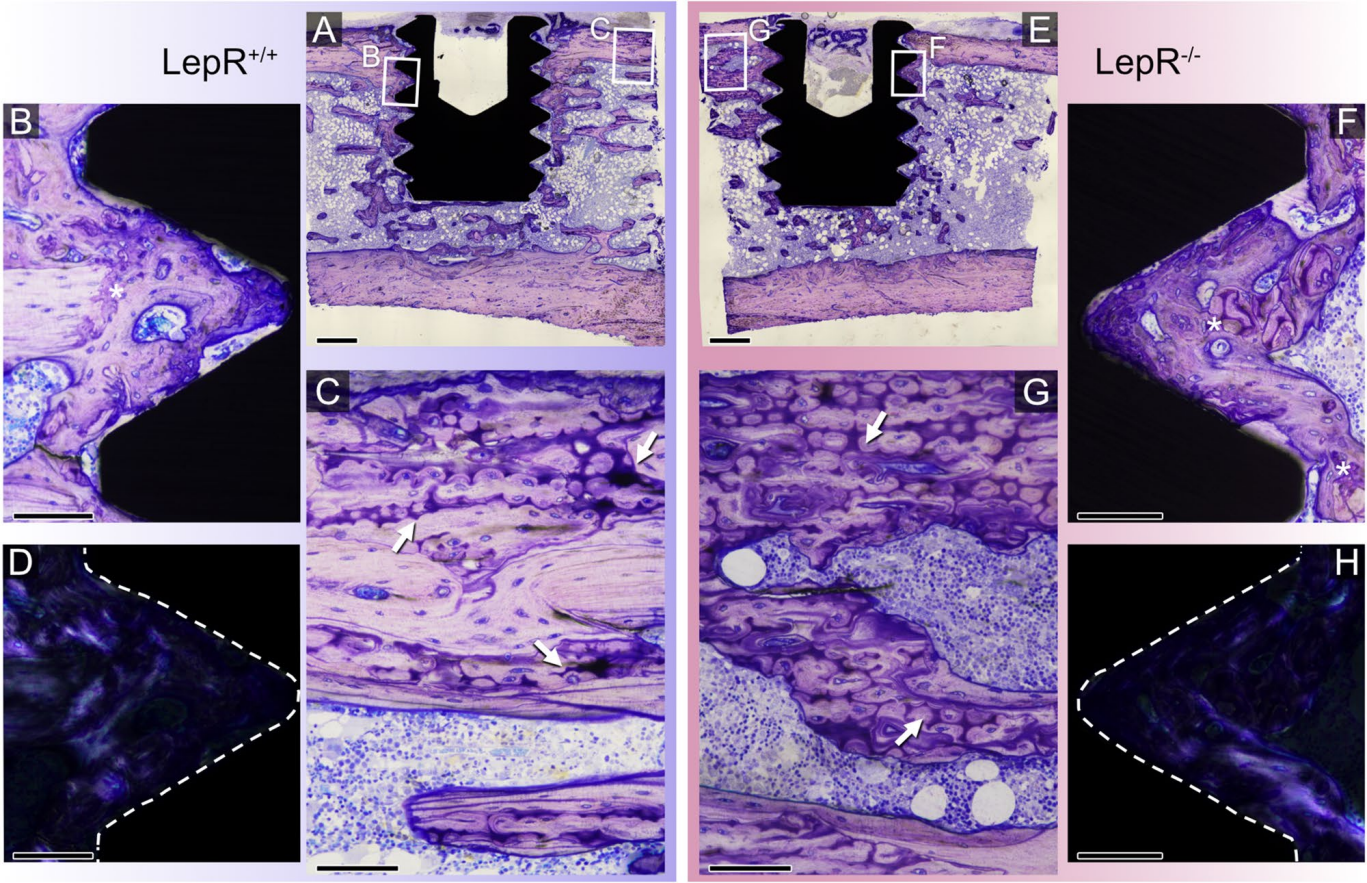
Qualitative evaluation of undecalcified basic fuchsin-stained histological sections of LepR^+/+^ (**A**–**D**) and LepR^−/−^ (**E**–**H**) animals. (**A**,** E**) Representative overview images of LepR^+/+^ and LepR^−/−^ sections showing unicortical placement of the machined Ti implants. Scale bars = 500 µm. (**C**,** G**) Large islands of leftover, hypermineralised cartilage (white arrows). Scale bars = 100 µm. (**B**,**F**) Recently formed bone is stained intensely blue. The interface between the native and de novo bone is easily discernible (white asterisks). Scale bars = 100 µm. (**D**,**H**) Polarised light microscopy of the corresponding threads in (**B**) and (**F**), respectively. Woven bone, due to its isotropic character does not transmit light, as observed in threads of both animal types. These areas correspond to areas of intensely stained, recently formed bone. Scale bars = 100 µm.

**Figure 6:**
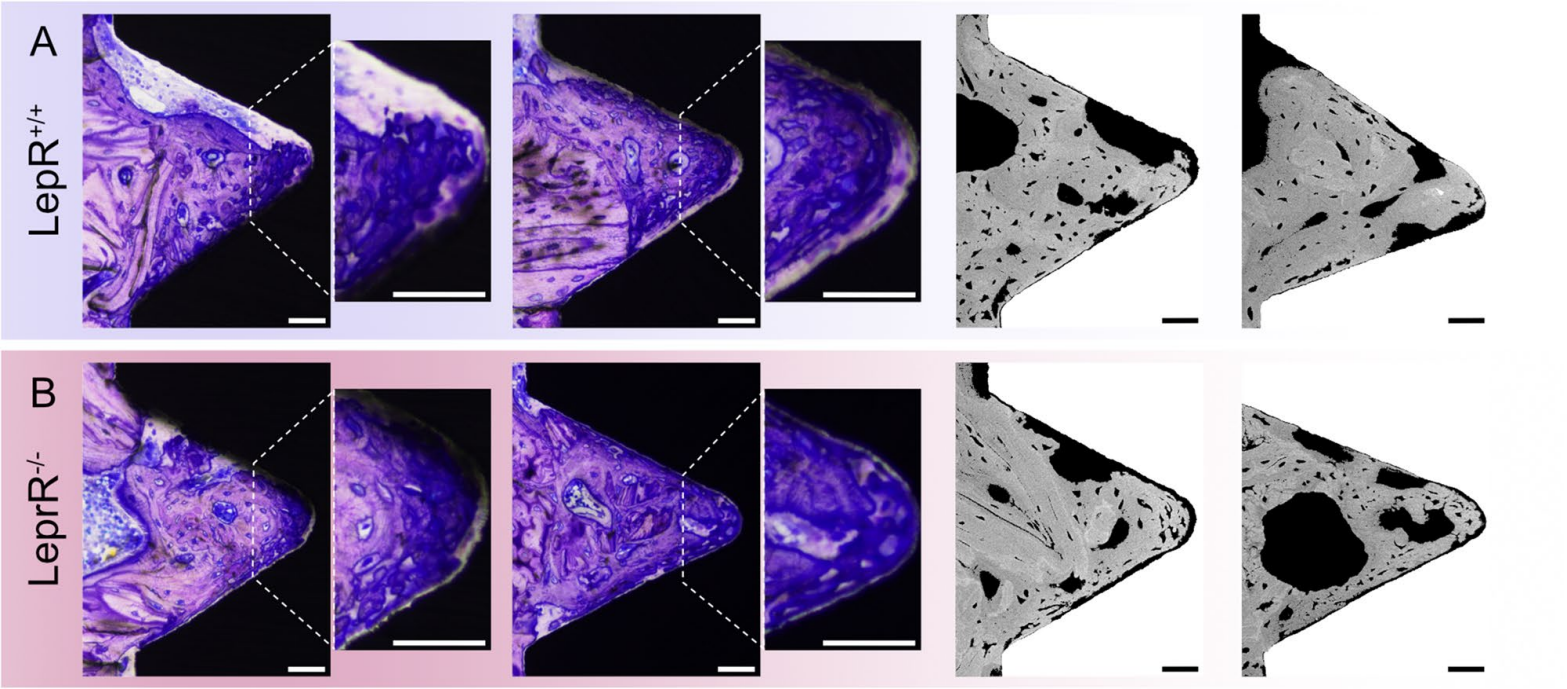
Newly formed bone within implant threads. Threads of (**A**) LepR^+/+^ and (**B**) LepR^−/−^ animals showing the presence of highly disorganised bone matrix as observed under light microscopy (left) and BSE-SEM (right). Scale bars = 50 µm.

Undecalcified basic fuchsin-stained histological sections were qualitatively assessed using brightfield optical microscopy (Fig. 5A–H). The most prominent features in both LepR^+/+^ and LepR^−/−^ sections were easily distinguishable islands of hypermineralised cartilage, interpreted to be remnants of the endochondral ossification (Fig. 5C, G). These islands were present in both cortical and trabecular bone and were devoid of cells, and upon qualitative evaluation, they were more prevalent in sections of LepR^−/−^ animals. The presence of immature, woven bone, as well as of more organised lamellar bone, suggests several bone-forming cycles (Fig. 5B, F) within the implant threads. The presence of newly formed, disorganised, woven bone was confirmed with polarised light microscopy (Fig. 5D, H). The initial stages of bone formation, both in LepR^+/+^ and LepR^−/−^ animals, were observed at 28 days of healing (Fig. 6A, B).

## Discussion

In this study, we investigated whether the influence of advanced metabolic syndrome, characterised by severe obesity, chronic hyperglycaemia, and developmental discrepancy^36^, translates to bone healing around biomaterials such as bone-anchored titanium implants^39–41^. Here, we used the Lund MetS rat with leptin receptor deficiency. LepR^−/−^ rats are obese as early as 4 weeks of age with severe type 2 diabetes mellitus developing in the animals by 21 weeks of age^30^, thus closely mimicking the disease environment of chronic metabolic syndrome.

During the 4 weeks of our study, LepR^−/−^ animals were on average ~40% heavier than the age-matched LepR^+/+^ animals, and blood glucose levels were more than doubled in the LepR^−/−^ rats, confirming the obese and hyperglycaemic features of the model. Secreted by osteocytes, sclerostin acts as an inhibitor of the Wnt/β-catenin signalling pathway and has been proven to reduce bone formation, bone volume, and bone strength in *SOST* knock-out mice^42^. We measured the sclerostin concentration in the serum of both animal groups on Day 0 and Day 28 of our study. In the hyperglycaemic LepR^−/−^ animals, sclerostin levels were substantially higher than in the euglycaemic LepR^+/+^ animals at both time points, which is in accordance with clinical findings in type 2 diabetes mellitus patients^43,44^. Evaluation of bone matrix composition revealed comparable BMDD parameters in the native bone of LepR^+/+^ and LepR^−/−^ animals, suggesting that the bone mineralisation patterns are not affected by leptin receptor deficiency. However, the mineral heterogeneity, represented by the Ca_WIDTH_, was significantly higher in the native bone of LepR^−/−^ animals. Moreover, the appearance of the averaged BMDD curve shows a higher area of highly mineralised bone matrix in LepR^−/−^ animals. Such areas of high Ca concentration correspond with the presence of leftover islands of highly mineralised cartilage in the metaphyseal bone of 20-week-old animals. Increased mineral heterogeneity and a larger area of highly mineralised bone in LepR^−/−^ animals point towards slower bone formation and/or delayed remodelling in these animals. Our findings are in agreement with previous observations in Zucker diabetic fatty fa/fa (ZDF) rats, where both a higher mineral heterogeneity and a higher fraction of highly mineralised bone matrix were observed^45^.

Within the implant threads of LepR^−/−^ animals, we found a reduced BV/TV. Impaired bone formation within the threads in these animals may be a consequence of increased sclerostin serum levels in LepR^−/−^ rats. Removal torque is often used in preclinical research to measure the direct physical interlocking of the implant in bone^46^, and a strong positive relationship between the BIC and RTQ measurements has been previously demonstrated in healthy animals^47^. However, changes in the extracellular matrix caused by disease and ageing can strongly affect the overall mechanical strength of bone^48^ and influence the stability of metal implants without affecting the extent of bone-implant contact. Moreover, different pharmacological agents, with direct and indirect influences on bone metabolism, can affect the biomechanical stability of implants without changing the BIC^38^. In studies using animal models of type 2 diabetes mellitus presenting with hyperglycaemia and obesity, a decrease in the BIC is often reported in the diabetic group^25,49^, seemingly reflecting a reduced osseointegration potential in these animals. Here, we measured an ~20% higher BIC in histological sections of LepR^−/−^ animals. However, the RTQ measurements of the implants of both LepR^−/−^ and LepR^+/+^ rats were comparable. Taken together, these findings indicate that bone in direct contact with the implant surface in LepR^−/−^ animals has less favourable mechanical properties. Nevertheless, the higher BIC compensates for the lower bone quality, yielding analogous biomechanical stability of the implants in LepR^−/−^ and LepR^+/+^ animals.

The mechanical properties of de novo and native bone in LepR^+/+^ and LepR^−/−^ animals were further assessed by characterisation of the extracellular matrix composition using Raman spectroscopy. The mineral crystallinity was consistent between the ROIs in both LepR^+/+^ and LepR^−/−^ animals, suggesting that the size and crystalline perfection of the apatite were not affected by leptin receptor deficiency. The lower mineral crystallinity in the native bone of LepR^+/+^ and LepR^−/−^ animals may be explained by the higher carbonate content. Carbonate substitution for phosphate (B-type substitution) in the apatite lattice correlates with decreased crystallinity^50,51^. Considering it as an indicator of relative tissue age^52^ and turnover, the significantly higher carbonate-to-phosphate ratio in the LepR^−/−^ animals suggests delayed bone remodelling. The mineral-to-matrix ratio was comparable between the LepR^+/+^ and LepR^−/−^ rats, with considerably lower values measured in the bone in close proximity to the implant. The mineral content, or mineral-to-matrix ratio as determined from vibrational spectroscopy, is a common indicator of bone mechanical properties on the micrometre scale^53^. A lower mineral-to-matrix ratio reveals that de novo bone within the implant threads is relatively younger and is expected to be less mechanically competent than native bone. However, importantly, this difference was much more pronounced in the LepR^−/−^ animals, where an ~90% higher mineral-to-matrix ratio was measured in the native bone.

To better understand the molecular processes in the peri-implant bone of LepR^+/+^ and LepR^−/−^animals, we investigated the gene expression of bone formation, bone remodelling, and proinflammatory markers. Despite the higher serum sclerostin levels in LepR^−/−^ animals, gene expression analysis of the peri-implant bone shows comparable relative gene expression of *SOST* between LepR^+/+^ and LepR^−/−^ animals. It appears that serum sclerostin levels need not necessarily correlate with *SOST* gene expression in bone. In Zucker rats, high serum sclerostin levels at 14 weeks of age could not be correlated with *SOST* gene expression or immunohistochemical labelling of sclerostin-positive osteocytes, which were comparable between the ZDF and control Zucker rats^54^. Given that the increased expression of *SOST* coupled with lowered expression of *RUNX2* can lead to impaired bone formatio ^55^, the absence of significant differences in our study suggests that at 28 days of healing, peri-implant bone formation has potentially reached a state of equilibrium in both LepR^+/+^ and LepR^−/−^ animals. In bone, BMP2 can have a twofold effect—bone formation by activating *RUNX2* expression required for osteoblast differentiation^56^ or bone resorption by stimulating osteoclastogenesis^57,58^. Significantly higher expression of *BMP2* in LepR^+/+^ animals may imply delayed initiation of the bone remodelling process in hyperglycaemic conditions. In implant-adherent cells of LepR^−/−^ animals, we found significantly higher expression of *RANK,* which points towards a higher prevalence of osteoclast progenitors on the surface of the implants in LepR^−/−^ animals. Future studies should incorporate gene expression analysis at earlier points of osseointegration to elucidate the signalling pathways involved in inflammation, osteogenesis, and bone remodelling in this model.

The major histological observation in both LepR^+/+^ and LepR^−/−^ animals is the areas reminiscent of hypermineralised cartilage, believed to be residuals of the endochondral ossification process^59^. The presence of such islands has been previously reported in ZDF rats^45^. Here, the higher prevalence of hypermineralised cartilage remnants in the sections of LepR^−/−^ animals may be related to the impaired longitudinal growth of long bones in these animals^36^, a feature also observed in ZF (Zucker fatty)^60^ and ZDF rats^61^. A recent study by Liddell et al.^62^ demonstrated continuous distal drift of the implants, with implants placed in the proximal tibial metaphysis of Wistar rats drifting to the diaphysis by 168 days of healing as a consequence of a normal endochondral ossification process at the epiphyseal growth plate. In our study, the implants placed in the metaphysis of the tibia remained in closer proximity to the epiphysial plate after 28 days of healing, which is explained by impaired longitudinal growth of long bones in LepR^−/−^ animals, as evident from an increased presence of islands of hypermineralised cartilage. Moreover, delayed bone remodelling caused by chronic hyperglycaemia in LepR^−/−^ rats might have exacerbated the prevalence of those islands compared to bone of LepR^+/+^ rats. Observations made in the de novo formed bone within implant threads were comparable between the LepR^+/+^ and LepR^−/−^ animals, suggesting similar healing potential in both animal groups. Interestingly, areas of highly disorganised woven bone adjacent to implants were distinguishable in both LepR^+/+^ and LepR^−/−^ rats. While such observations are usually made at early healing stages (e.g., 6 days), woven bone is fully remodelled by 28 days of healing in Sprague‒Dawley rats^63^ indicating that observation of woven bone at day 28 in both LepR^+/+^ and LepR^−/−^ animals could be due to a shared genetic background and not affected by leptin receptor deficiency.

There is ongoing debate whether obesity and hyperglycaemia are definite contraindications for successful implant treatment. Likewise, there is a lack of conclusive negative impact on the survival of dental implants despite studies in both obese and diabetic patients showing greater marginal bone loss^23,64^. In accordance with these findings, we show that osseointegration did not differ between LepR^−/−^ and LepR^+/+^ animals, suggesting that these conflicting clinical outcomes might be related to discreet effects of hyperglycaemia and obesity on bone healing around metal implants. However, given that our study was performed in an animal model care should be taken when translating these findings to clinical conditions.

## Conclusions

This study offers a comprehensive evaluation of osseointegration in a novel animal model of metabolic syndrome. Overall osseointegration was comparable at 28 days of healing in LepR^−/−^ and LepR^+/+^ animals. However, the low bone volume coupled with the high bone-to-implant contact and comparable biomechanical stability of the implants point to changed bone formation, remodelling, and bone quality properties in LepR^−/−^ animals. This observation was corroborated by the significant difference in the carbonate-to-phosphate ratio between the native and de novo bone in LepR^−/−^animals. One of the limitations of this animal model is the early onset of hyperglycaemia, taking place before the skeletal maturity of rats at 16 weeks of age^65^, meaning that even at 16 weeks of age (age of the animals at the beginning of our study) animals may have not reached skeletal maturity. Moreover, the duration of the disease could influence bone quality and turnover due to cumulative negative effects of prolonged hyperglycaemia that can lead to micro- and macro-vasculature complications, and damage to cardiovascular, and urinary systems. Given the age of the animals used here, further studies are needed to elucidate the effects of long-term hyperglycaemia and obesity on osseointegration. Additionally, evaluation of earlier time points could offer more insight into the early osseointegration processes, which may be more prominently affected.

## Materials and methods

### In vivo animal model, surgery, and sample processing

The influence of the defective leptin receptor gene on healing and osseointegration was evaluated using the Lund MetS rat model (Janvier Labs, France). The animal model was developed by introgression of the defective leptin receptor gene of Koletsky rats into BioBreeding Diabetes Resistant (BBDR) rats, resulting in a congenic BBDR.cg-*Lepr*^*db/db*^*.cp*/LundRj rat line^35^. Two distinct groups of male rats, hyperglycaemic homozygous (LepR^−/−^) and normoglycaemic homozygous (LepR^+/+^) animals, received machined, screw-shaped, commercially pure titanium implants in the proximal metaphysis of each tibia.

A total of fourteen 16-week-old animals (*n* = 7 per group) were included in the study. Surgery was performed under general anaesthesia (isoflurane inhalation) under sterile conditions. In brief, the medial side of the proximal tibial metaphysis was exposed, and the implantation site was prepared successively with low-speed round burs of 1.6- and 1.8-mm diameter under constant irrigation with saline. Following implant insertion, the surgical wounds were closed with polyglactin (Vicryl Rapide™, Ethicon Inc., USA) for internal sutures and poliglecaprone (Monocryl^®^, Ethicon Inc., USA) for transcutaneous sutures. All animals received subcutaneous injections of buprenorphine analgesic (Temgesic^®^, Reckitt Benckiser Healthcare Limited, UK) before and after surgery to alleviate pain. All animals had ad libitum access to food and water throughout the study, and no restrictions on their movement were imposed. After 28 days of healing, the rats were placed under general anaesthesia and euthanised with an intraperitoneal overdose of sodium pentobarbital (60 mg/ml).

Immediately upon sacrifice, the implant heads were exposed, and the RTQ was measured in either the left or right tibial implants with a constant angular speed of 0.2°/s in a randomised order. Once the RTQ was measured, the implants were carefully unscrewed and collected for gene expression analysis of implant-adherent cells as described previously^66^. The leftover surrounding bone was dissected with a trephine bur and preserved for further gene expression analysis. Opposing tibial implants, with the intact bone-implant interface, were dissected *en bloc* with the surrounding bone, placed in 10% neutral buffered formalin, dehydrated in a graded ethanol series, and resin embedded (LR White Resin, London Resin Co. Ltd., UK) for additional analyses. The body weight and blood glucose levels of animals were monitored at implantation (Day 0) and the endpoint (Day 28). Random blood glucose tests were performed before the surgical procedures on Day 0 and 28 with Accu-Chek^®^ Aviva (Roche Diabetes Care, USA) to confirm hyperglycaemia and euglycemia in LepR^−/−^ and LepR^+/+^ animals, respectively. Further blood samples were taken for serum collection at both time points.

### Immunological assay

Whole blood samples were collected from the jugular vein of all animals at the time of implantation and the experimental endpoint while the animals were under general anaesthesia. Blood was allowed to clot at room temperature for 60 min and then was centrifuged for 10 min at 2000×*g*. After serum and blood clot separation, the serum was collected, aliquoted to avoid repeated freeze‒thaw cycles, and stored at −80 °C until further processing. Enzyme-linked immunosorbent assay (ELISA) was used to measure the concentration of sclerostin in rat serum using a Quantikine^®^ ELISA Mouse/Rat SOST immunoassay kit (R&D Systems^®^, Bio-Techne Corporation, USA) per the manufacturer’s instructions. The optical density of each sample was detected using a FLUOstar^®^ Omega microplate reader (BMG LABTECH, Germany) set to 450 and 540 nm wavelengths. Using a standard curve and linear regression analysis, the optical density was translated into the sclerostin concentration in serum samples at two time points.

### X-ray micro-computed tomography and histology

Following the embedding procedure, the bone volume within the implant threads was measured using micro-CT (Skyscan 1172, Bruker, Belgium). Scans were made at 100 kV acceleration voltage and 100 µA current, while low-energy X-rays were blocked with a Cu (40 µm) and Al (0.5 mm) filter. The imaging resolution was 11.76 µm. Reconstruction of the images was performed in NRecon software v.1.6.9.8 (Bruker, Belgium), and data analysis was performed using CTan software v.1.20.8.0+ (Bruker, Belgium). The bone volume (BV/TV) was measured in the ROI demarcated within the threads of the implant. After micro-CT evaluation, resin bone-implant blocks were bisected. One half was used in the preparation of undecalcified, ~40 µm thick, ground sections (EXACT cutting and grinding equipment, EXACT Advanced Technologies GmbH, Germany) and stained with basic fuchsin. Brightfield imaging was performed on a Nikon Eclipse E600 optical microscope (Nikon Ltd., Japan) to determine the percentage of BIC, the percentage of B.Ar filling the thread, and to perform a qualitative histological evaluation of the samples.

### Quantitative backscattered electron imaging

The other halves of the resin-embedded bone-implant blocks were used for qBEI to evaluate the BMDD. Backscattered electron scanning electron microscopy (BSE-SEM) was performed in a Quanta 200 environmental SEM (FEI Company, The Netherlands) under a 20.0 kV accelerating voltage, 1 Torr water vapour pressure, and a working distance of 10 mm, with magnification set at 200× (0.63 µm). The brightness and contrast were adjusted before imaging to appropriate grey-level values of aluminium-carbon standards, at 225 for Al and 25 for C. Standards were made using standard aluminium SEM specimen stubs (Agar Scientific Ltd., UK) and graphite rods (Alfa Aesar™, Thermo Fisher Scientific, USA**)** embedded in LR White Resin (London Resin Co. Ltd., UK). Both the samples and standards were wet-polished using 400–4000 grit SiC paper and absolute ethanol.

Using calibrated, grey-level images, ROIs of 5–7 mm^2^ (depending on the availability of bone) were selected in the native bone of both groups. Demarcation of ROIs and generation of histograms were performed using ImageJ (*imagej.nih.gov/ij*). The relationship between the grey-level values and atomic number was calculated using images of the standards. The grey-level value of each pixel represents the calcium concentration (Ca [wt.%]). These values are used to determine the Ca_MEAN_—weighted mean of Ca [wt.%], Ca_PEAK_—most frequently observed Ca [wt.%], and Ca_WIDTH_—mineral heterogeneity calculated as the full-width at half-maximum under the bone mineralisation density distribution curves.

### Raman spectroscopy

The composition of native and newly formed bone within the implant thread was probed using a confocal Raman microscope (Renishaw inVia Qontor, Renishaw plc. Wotton under Edge, UK) Measurements were made with a 633 nm laser focussed through a 100×/0.9 NA objective, and the Raman scattered light was collected using a Peltier-cooled charge-coupled device deep depletion near-infrared detector behind an 1800 g mm^−1^ grating. Seven point measurements were taken within the thread of the implant, keeping within 20 µm from the implant surface at 8 s integration time and 5 accumulations. The corresponding native bone was measured using the same acquisition parameters. Using Spectragryph V1.2.16.1^67^, the acquired Raman spectra were processed by performing background subtraction using an adaptive baseline set to 10% coarseness, followed by cosmic ray removal using the remove spikes function and maximum width of spikes set to 5 pixels.

### Gene expression

Implants with attached bone tissue (implant-adherent cells) were placed in nucleic acid stabilisation reagent (DNA/RNA Shield, Zymo Research, USA) and kept at −80 °C until RNA isolation. Samples of implant-adherent cells were homogenised in RLT buffer with β-mercaptoethanol using a TissueLyzer II instrument (Qiagen, Germany), centrifuged at 16,000×*g* for 3 min, and total RNA was isolated using an RNeasy^®^ Micro Kit (Qiagen, Germany) per the manufacturer’s instructions. Trephine excised bone samples were preserved in an RNA*later*™ Stabilization Solution (Invitrogen™, Thermo Fisher Scientific, USA) for 24 h at 4 °C and transferred to −80 °C until further processing. Mechanical tissue disruption was performed in TRIzol™ Reagent (Invitrogen™, Thermo Fisher Scientific, USA) using steel beads and a TissueLyzer II (Qiagen, Germany) for 3.5 min at 25 Hz. Cold liquid-phase separation was used for total RNA extraction, and RNA purification of the aqueous phase was performed using an RNeasy^®^ Mini Kit (Qiagen, Germany) following the manufacturer’s instructions. Quantification of RNA isolated from bone and implant-adherent cells was performed with a DeNovix RNA Assay (DeNovix Inc., USA), and RNA quality assessment was evaluated using an Agilent 6000 RNA Nano Kit (Agilent Technologies Inc., USA). Reverse transcription of implant-adherent cells and bone RNA was performed with a GrandScript cDNA synthesis kit (TATAA Biocentre AB, Sweden).

Gene expression analysis included genes coding for sclerostin (SOST), receptor activator of nuclear factor-kappa B (RANK) and its ligand (RANKL), osteoprotegerin (OPG), peroxisome proliferator-activated receptor gamma (PPARG), Runt-related transcription factor 2 (RUNX2), bone morphogenetic protein 2 (BMP2), and finally, tumour necrosis factor alpha (TNFa).

Screening for appropriate reference genes in both sample types (implant-adherent cells and bone) was performed on genes coding for β-glucuronidase (GUSB), glyceraldehyde-3-phosphate dehydrogenase (GAPDH), β-actin (ACTB), hypoxanthine phosphoribosyltransferase 1 (HPRT1), tubulin β polypeptide (TUBB), and tyrosine 3-monooxygenase/tryptophan 5-monooxygenase activation protein ζ (YWHAZ). All genes selected for reference gene screening are included in the commercial reference gene panel (TATAA Biocenter AB, Sweden). Reference gene selection was performed using geNorm^68^ and NormFinder^69^ software, and *GAPDH* was chosen as the reference gene for both implant-adherent cells and bone tissue samples.

Gene expression was performed using a QX200 ™ AutoDG Droplet Digital™ PCR System (Bio-Rad Laboratories, USA). Differences in the amplicon length of selected genes of interest allowed for the design of two-target, EvaGreen-based ddPCR assays as described previously by others^70^. The ddPCR master mix consisted of a QX200™ ddPCR™ EvaGreen Supermix (2×) (Bio-Rad Laboratories, USA), forward and reverse primers of appropriate gene targets (Integrated DNA Technologies, Inc., USA), and DNase/RNase free H_2_O. Finally, the cDNA of interest was added before thermal cycling in a C1000 Touch™ Thermal Cycler (Bio-Rad Laboratories, USA). The fluorescence of droplets was measured using ddPCR™ Droplet Reader Oil (Bio-Rad Laboratories, USA) in a QX100/200™ Droplet Reader (Bio-Rad Laboratories, USA). Data were analysed in QuantaSoft™ Software v.1.7.4.0917. Exact primer sequences, assay pairings, and thermocycling conditions are given in the Supplementary Information.

### Statistical analysis

All statistical analyses were performed using GraphPad Prism v9.4.1 (GraphPad Software, USA). Comparisons between the two groups were made with a nonparametric Mann‒Whitney test. Data are presented as the mean value with standard deviation (± SD). P values of < 0.05 were considered statistically significant.

### Ethical approval

The animal study was approved by the Animal Research Ethics Committee of Gothenburg, Sweden (DNr. 14,790/2019) and were conducted in full compliance with the Directive 2010/63/EU, the national regulations, and ARRIVE guidelines.

### Supplementary Information

Supplementary Information.

## Data Availability

The datasets used and/or analysed during the current study are available from the corresponding author on reasonable request.
