# Supplementary material for: Leptin receptor gene deficiency minimally affects osseointegration in rats
Source: Sci Rep. 2023 Sep 20;13:15631. doi: 10.1038/s41598-023-42379-5 (PMC10511412; doi:10.1038/s41598-023-42379-5)
Supplement: Supplementary file 1 — Supplementary Information. [file 41598_2023_42379_MOESM1_ESM.pdf]

Supplementary Information:

Leptin receptor gene deficiency minimally affects osseointegration in rats

Martina Jolic[1], Krisztina Ruscsák[1], Lena Emanuelsson[1], Birgitta Norlindh[1], Peter Thomsen[1], Furqan A. Shah[1], and Anders Palmquist[1,*]

[1] Department of Biomaterials, Institute of Clinical Sciences, Sahlgrenska Academy, University of Gothenburg, Gothenburg, Sweden

*Corresponding author:

Supplement 1.

Primer sequences:

Gene	Direction	Primer sequence
RUNX2 (Runt-related transcription factor 2)	FWD	5’- CGAAATGCCTCTGCTGTT -3’
	REV	5’- AAAGTGAAACTCTTGCCTCGT -3’
RANKL (Ligand of receptor activator of NF-κB)	FWD	5’- AGAGCGAAGACACAGAAGCA -3’
	REV	5’- GAGCCACGAACCTTCCATCA -3’
RANK (Receptor activator of NF-κB)	FWD	5’- GATGGAAGCACACCAAGGGA -3’
	REV	5’- CAGCCACCACTACCACAGAG -3’
OPG (Osteoprotegrin)	FWD	5’- TCGAAAGCACCCTGTAGGAA -3’
	REV	5’- AGGCAAACTGTCCACCAGAA -3’
SOST (Sclerostin)	FWD	5’- TACATGCAGCCTTCGTTGCT -3’
	REV	5’- GCTGTACTCGGACACGTCTTT -3’
PPARG (Peroxisome proliferator-activated receptor gamma)	FWD	5’- GCTTGTGAAGGATGCAAGGG -3’
	REV	5’- GCCCAAACCTGATGGCATTG -3’
BMP2 (Bone morphogenetic protein 2)	FWD	5’- ATATGCTCGACCTGTACCGC -3’
	REV	5’- TTGAAGAAGAAGCGTCGGGA -3’
TNFa (Tumour necrosis factor alpha)	FWD	5’- GGGCTCCCTCTCATCAGTTC -3’
	REV	5’- CCGCTTGGTGGTTTGCTAC -3’

Assay pairs, amplicon length:

Assay pair	Amplicon lengths
RUNX2 – PPARG	92 bp – 172 bp
RANKL – OPG	143 bp – 219 bp
BMP2 – TNFa	172 bp – 106 bp
SOST – RANK	197 bp – 143 bp

*bp = base pairs

Thermocycling conditions for all assays:

Step	Temperature (°C)	Time	Ramp rate	Number of Cycles
Enzyme activation	95	5 min	2 °C/s	1
Denaturation	95	30 s		40
Annealing/extension	61	1 min		40
Signal stabilisation	4	5 min		1
	90	5 min		1
Hold	4	∞		1
